# Forced Magnetostrictions and Magnetizations of Ni_2+*x*_MnGa_1−*x*_ at Its Curie Temperature

**DOI:** 10.3390/ma11112115

**Published:** 2018-10-28

**Authors:** Takuo Sakon, Yuhi Hayashi, Dexin Li, Fuminori Honda, Gendo Oomi, Yasuo Narumi, Masayuki Hagiwara, Takeshi Kanomata, Tetsujiro Eto

**Affiliations:** 1 Department of Mechanical and System Engineering, Faculty of Science and Technology, Ryukoku University, Otsu, Shiga 520-2194, Japan; t150289@mail.ryukoku.ac.jp; 2 Institute for Materials Research, Tohoku University, Oarai, Ibaraki 311-1313, Japan; dxli@imr.tohoku.ac.jp (D.L.); honda@imr.tohoku.ac.jp (F.H.); 3 Kurume Institute of Technology, Kurume, Fukuoka 830-0052, Japan; geomi@kurume-it.ac.jp (G.O.); teto@kurume-it.ac.jp (T.E.); 4 Center for Advanced High Magnetic Field Science, Graduate School of Science, Osaka University, 1-1 Machikaneyama, Toyonaka, Osaka 560-0043, Japan; narumi@ahmf.sci.osaka-u.ac.jp (Y.N.); hagiwara@ahmf.sci.osaka-u.ac.jp (M.H.); 5 Research Institute for Engineering and Technology, Tohoku Gakuin University, Tagajo, Miyagi 985-8537, Japan; kanomata@mail.tohoku-gakuin.ac.jp

**Keywords:** ferromagnetic Heusler alloy, magnetization, magnetostriction, itinerant electron magnetism, premartensite phase

## Abstract

Experimental investigations into the field dependence of magnetization and the relationship between magnetization and magnetostriction in Ni_2+*x*_MnGa_1−*x*_ (*x* = 0.00, 0.02, 0.04) alloy ferromagnets were performed following the self-consistent renormalization (SCR) spin fluctuation theory of itinerant ferromagnetism. In this study, we investigated the magnetization of and magnetostriction on Ni_2+*x*_MnGa_1−*x*_ (*x* = 0.02, 0.04) to check whether these relations held when the ratio of Ni to Ga and, the valence electron concentration per atom, *e*/*a* were varied. When the ratio of Ni to Ga was varied, *e*/*a* increased with increasing *x*. The magnetization results for *x* = 0.02 (*e*/*a* = 7.535) and 0.04 (*e*/*a* = 7.570) suggest that the critical index *δ* of *H* ∝ *M^δ^* is around 5.0 at the Curie temperature *T*_C_, which is the critical temperature of the ferromagnetic–paramagnetic transition. This result confirms Takahashi’s spin fluctuation theory and the experimental results of Ni_2_MnGa. The spontaneous magnetization *p*_S_ slightly decreased with increasing *x*. For *x* = 0.00, the spin fluctuation parameter in *k*-space (momentum space; *T*_A_) and that in energy space (*T*_0_) were obtained. The relationship between *p*_eff_/*p*_S_ and *T*_C_/*T*_0_ can also be explained by Takahashi’s theory, where *p*_eff_ indicates the effective magnetic moments. We created a generalized Rhodes-Wohlfarth plot of *p*_eff_/*p*_S_ versus *T*_C_/*T*_0_ for other ferromagnets. The plot indicates that the relationship between *p*_eff_/*p*_S_ and *T*_0_/*T*_C_ follows Takahashi’s theory. We also measured the magnetostriction for Ni_2+*x*_MnGa_1−*x*_ (*x* = 0.02, 0.04). As a result, at *T*_C_, the plot of the magnetostriction (Δ*L*/*L*) versus *M*^4^ shows proportionality and crosses the origin. These magnetization and magnetostriction results were analyzed in terms of Takahashi’s SCR spin fluctuation theory. We investigated the magnetostriction at the premartensite phase, which is the precursor state to the martensitic transition. In Ni_2_MnGa system alloys, the maximum value of magnetostriction is almost proportional to the *e*/*a*.

## 1. Introduction

Spin fluctuation theories have advanced the attempts to elucidate the physical principles of the itinerant electron system [1,2,3,4,5]. According to the self-consistent renormalization (SCR) spin fluctuation theory [1], the external magnetic field *H* is proportional to the third power of the magnetization *M*^3^ at the Curie temperature *T*_C_. This relation was derived by only considering the transverse modes of the thermal spin fluctuations with respect to the direction of the static and uniform magnetic moment [6,7]. Takahashi proposed SCR theory according to zero-point spin fluctuations, which assimilate both the transverse and the longitudinal components of the fluctuations [3,4,5,8]. An outstanding characteristic of this theory is the magnetization at *T*_C_. This theory proposed by Takahashi indicates that *H* is proportional to *M*^5^ at *T*_C_.

The thermo-dynamical relationship between the magnetization *M* and the external magnetic field *H* can be expressed by the equation:

(1) $$H=\frac{\partial F}{\partial M}=a\left(T\right)M+b\left(T\right)M^{3}+c\left(T\right)M^{5}+\cdots$$

where *F* indicates the spin fluctuation free energy. This appears as Equation (2.59) in Takahashi [8].

As *T*→*T*_C_, the magnetic susceptibility χ(T) comes infinite. Therefore,

(2) $$\underset{T\to T_{C}}{\lim}a\left(T\right)=\underset{T\to T_{C}}{\lim}\frac{H}{M}=\underset{T\to T_{C}}{\lim}\frac{1}{\chi }=0$$

Then, the first expansion coefficient at *T*_C_ is *a*(*T*_C_) = 0.

According to the Rhodes-Wohlfarth theory [9], the third expansion coefficient *b*(*T*) in Equation (1) remains finite at *T* = *T*_C_. Therefore, the following formula is satisfied at *T*_C_:

(3) $$H=b(T_{C})M^{3}+c(T_{C})M^{5}+\cdots$$

Under the Takahashi theory, *b*(*T*) vanishes at *T*_C_, as shown in Equation (3.51) in Takahashi [8].

As a result, the *M* dependence of the magnetic fields *H* can be explained by the equation:

(4) $$H=c(T_{C})M^{5}$$

In Equation (4), higher terms are ignored because their magnitudes are smaller than that of the third term. In conclusion, an *H* ∝ *M*^5^ relation was obtained.

MnSi [3], CoS_2_ [10], Fe*_x_*Co_1−*x*_Si [11], and Ni [12] follow the relationship provided in Equation (4). The Heusler isotropic ferromagnetic alloy Ni_2_MnGa also follows this relation in a cubic austenite phase [12]. For Ni_2_MnGa, the critical index *δ* of *H* ∝ *M^δ^* at *T*_C_ is *δ* 4.70 ± 0.5 [12,13].

Takahashi proposed that magnetostriction can be observed due to the itinerant spin fluctuations around *T*_C_ [8] because the magnetostriction is calculated from the spin fluctuation free energy. The relationship between the magnetostriction and the magnetization at *T*_C_ [8] in Equation (6.101) was explained using the formula

(5) $$\frac{\omega_{h}\left(\sigma ,t_{C}\right)}{\omega_{0}}=K\times A\left(0,t_{C}\right)\times \frac{\sigma^{4}}{\sigma_{0}^{4}}$$

where *t*_C_ is a relative Curie temperature; σ and *σ*_0_ are the magnetization in a magnetic field and the spontaneous magnetization, respectively; *ω*_0_ is the nonmagnetic volume contribution; *w*_h_(σ, *t*_C_) is the relative magnetic volume-striction at *T*_C_; *K* has a constant value in an isothermal state; and *A*(0, *t*_C_) indicates the amplitude of the thermal spin fluctuations at *T*_C_. Equation (5) indicates that the magnetostriction is proportional to *M*^4^ at *T*_C_. Kittel mentioned that the volume strain Δ*V*/*V* is three times the value of Δ*L*/*L* [14]. Accordingly, volume magnetostriction (Δ*V*/*V*) discussions were applied to the results of the magnetostriction Δ*L*/*L* in this experimental study. 

For quondam research, an investigation into MnSi, which is famed for its weak itinerant magnetism, was completed [15]. The magnetostriction Δ*L*/*L* versus the square of the magnetization *M*^2^ was analyzed. Around *T*_C_ = 30 K, the plot strayed from linearity. Takahashi proposed that around *T*_C_, the magnetostriction is not proportional to the square of the magnetization. Δ*L*/*L* is proportional to *M*^4^ through the origin at *T* = 29 K around *T*_C_ [8]. In a previous study, we investigated the magnetostriction property of a polycrystalline Ni_2_MnGa alloy using the self-consistent renormalization (SCR) theory of itinerant ferromagnets [13]. The magnetostriction was found to be proportional to the fourth power of magnetization. At the Curie temperature, magnetostriction crossed the point of origin. These results are in line with Takahashi’s spin fluctuation theory. In this study, we investigated Ni_2+*x*_MnGa_1−*x*_ (*x* = 0.02, 0.04) alloys and studied the effect of varying alloy composition (ratio of Ni and Ga atoms) on magnetostriction. We found that the valence electron concentration per atom, i.e., the ratio *e*/*a*, increases with increasing *x*. The *e*/*a* values were 7.50, 7.535, and 7.570 for *x* = 0.00, 0.02, and 0.04, respectively. The spin fluctuation parameter in wave number space (momentum space) *T*_A_ and that in energy space *T*_0_ were obtained from the results of the magnetization measurement. We discuss the relation between *p*_eff_/*p*_S_ and *T*_C_/*T*_0_ compared with that shown in other itinerant ferromagnets by means of a generalized Rhodes-Wohlfarth plot [8]. We also investigated the *e*/*a* dependences of the maximum magnetostriction around the premartensitic–austenitic transition for Ni_2_MnGa-type alloys. Researchers have studied the correlation between magnetostriction and the valence electron concentration *e*/*a*, which is related to the energy of the electron system [16,17,18]. In our prior study, we measured the properties of Ni_2_Mn_1−*x*_Cr*_x_*Ga [16]. In these alloys, the *e*/*a* was smaller than 7.50, which is the value for Ni_2_MnGa. In this study, we measured Ni_2+*x*_MnGa_1−*x*_ (*x* = 0.02, 0.04) alloys for which the *e*/*a* is larger than 7.50 and investigated the *e*/*a* dependence of the maximum magnetostriction in the premartensite phase.

Rizal et al. investigated the magnetic property of nanostructured Fe-Co alloys [19]. At room temperature, a strong correlation was found between the saturated magnetization and the lattice constant of the Fe-Co alloy. For Ni_2_MnGa-type Heusler alloys, the correlations between *e*/*a* and the magnetization (magnetic moment) or magnetostriction have been the subject of several investigations undertaken by varying alloy composition. Accordingly, in this article, we focused on the *e*/*a* dependences of the magnetostrictions.

## 2. Materials and Methods

The polycrystalline samples of Ni_2+*x*_MnGa_1−*x*_ (*x* = 0.00, 0.02, 0.04) were prepared by arc melting the constituent elements—4N Ni, 3N Mn, and 6N Ga—several times in an Ar atmosphere. Each ingot was melted several times in order to ensure good homogeneity. The products from the arc melting process were sealed in an evacuated silica tube and solution heat-treatments were applied at 1123 K for three days. After these treatments, the sample was quenched in water. The measurement of permeability was performed in alternating current (AC) magnetic fields with a frequency of 73 Hz and a maximum field of ±10 Oe. The AC magnetic fields were measured using a gaussmeter 410 (Lakeshore Cryotronix Inc., Westerville, OH, USA). The sample size chosen for the experimental investigations was 3.0 × 3.0 × 4.0 mm. The magnetostriction was measured by means of a strain gauge [13]. The magnetostriction Δ*L*/*L* was measured parallel to the external magnetic field *H*—the same approach used in the experimental investigation of MnSi [15]. A helium-free superconducting magnet at the Center for Advanced High Magnetic Field Science, Osaka University, Japan was used for the magnetostriction measurements up to 5 T.

The magnetization measurements were performed using a solenoid-type pulsed-field magnet at Ryukoku University, Japan [13]. The absolute value of the magnetization was calibrated with the use of a sample of pure Ni of the same size. The same bulk sample was used in the permeability, magnetization, and magnetostriction measurements in order to compare the results. The data for magnetostriction and magnetization were the results of measurements with increasing magnetic fields beginning with a zero field. 

We also used a water-cooled magnet in a steady field up to 1.6 T, which was installed in Ryukoku University, and studied the magnetostriction in order to investigate the temperature dependence of the magnetostriction around the premartensite phase.

## 3. Results and Discussions

### 3.1. Magnetic Field Dependence of the Magnetization

**Figure 1:**
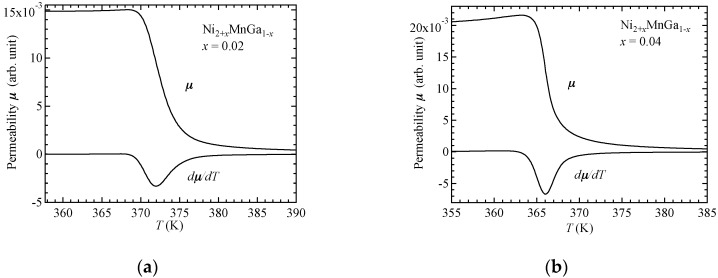
Plots of *μ* vs. *T* and *dμ*/*dT* vs. *T* for (**a**) *x* = 0.02 and (**b**) *x* = 0.04.

For the Ni_2_MnGa alloy, martensitic transitions occurred at the temperature *T*_M__S_ of 195 K [16]. The alloy Ni_2_MnGa also has a premartensite phase. This is a precursor (intermediate) state to the martensitic transition. In the premartensite phase, the alloy has a 3M modulated structure [20]. The austenitic–premartensitic transition occurs at the premartensitic temperature *T*_P_ of 260 K. Above *T*_P_, a cubic L2_1_ type austenite phase is realized. The Curie temperature *T_C_* is 375 K, which is much higher than *T*_MS_ and *T*_P_. The ferromagnetic–paramagnetic transition at *T_C_* occurs in the cubic austenite phase, and the magnetic anisotropy constant *K*_1_ in the austenite phase is 1/10 smaller than that in the martensite phase. The *K*_1_ value at 150 K in the martensite phase was of the magnitude 4.0 × 10^6^ erg/cm^3^, and *K*_1_ at 293 K in the austenite phase was 0.30 × 10^6^ erg/cm^3^ [16]. The magnitude of *K*_1_ of Ni_2_MnGa in the austenite phase is comparable to that of Fe. Therefore, Ni_2_MnGa was decided to be an isotropic ferromagnet in the austenite phase. The value of *T*_M_ for Ni_2+*x*_MnGa_1__−*x*_ increased with increasing Ni concentration *x*. The value of *T*_P_ also increased with increasing *x* for *x* ≤ 0.04. Above *T*_P_ = 265 K for *x* = 0.02 and 275 K for *x* = 0.04, a cubic L2_1_ type austenite phase is realized. Figure 1 plots the permeability *μ* for *x* = 0.02 (Figure 1a) and *x* = 0.04 (Figure 1b) during heating in a zero external magnetic field. The derivative of *μ* with respect to temperature, *dμ*/*dT*, is also shown in Figure 1. The *T*_C_ could not be defined from the *μ*-*T* curve because the divergence derived from Equation (2) was not found. Therefore the *T*_C_ was defined as a temperature where the absolute value of the gradient of the *μ*-T curve, *dμ*/*dT* is maximum. The Curie temperatures *T_C_* were found to be 372 K and 366 K for *x* = 0.02 and 0.04, respectively, as obtained from the peaks of *dμ*/*dT* in Figure 1. 

**Figure 2:**
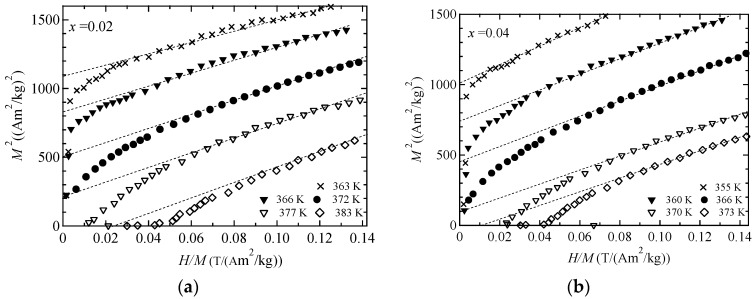
The *H*/*M* dependences of *M*^2^ for (**a**) *x* = 0.02 and (**b**) *x* = 0.04. The dotted straight lines are included as a visual guide.

**Figure 3:**
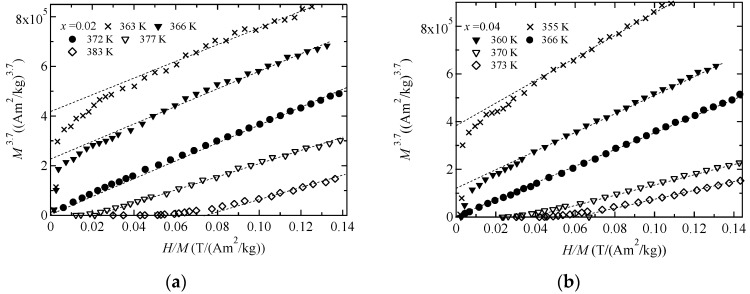
The *H*/*M* dependences of *M*^3.7^ for (**a**) *x* = 0.02 and (**b**) *x* = 0.04. The dotted straight lines are included as a visual guide.

**Figure 4:**
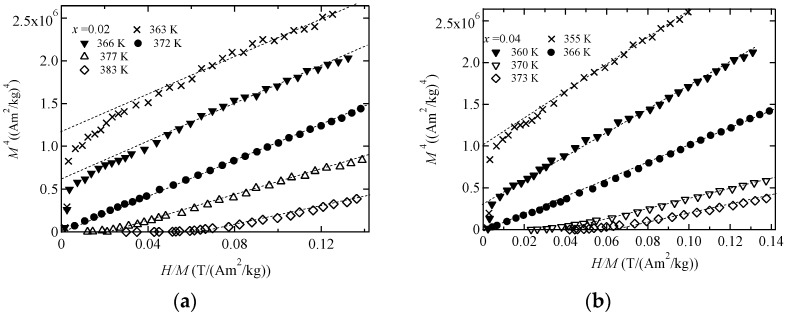
The *H*/*M* dependences of *M*^4^ for (**a**) *x* = 0.02 and (**b**) *x* = 0.04. The dotted straight lines are included as a visual guide.

We measured the magnetization of Ni_2+*x*_MnGa_1−*x*_ around *T_C_* for the purpose of ascertaining the critical index *δ* of *M^δ^*^−1^ versus *H*/*M*. We plotted figures of *M^δ^*^−1^ versus *H*/*M* for *δ =* 3.0, 4.7, and 5.0; these are shown in Figure 2, Figure 3 and Figure 4, respectively. The result for *δ =* 3 is comparable to Moriya’s theory [1], that for *δ* = 5 is comparable to Takahashi’s theory [8], and that for *δ* = 4.7 is comparable to the former result [12]. *M^δ^*^−1^ versus *H*/*M* with *δ* = 4.7 in Figure 3 and *δ* = 5.0 in Figure 4 show good linearity through the origin at *T*_C_, denoted by the filled circles. The results suggest that for *x* = 0.02 and 0.04, the critical index *δ* is 4.7–5.0, which conforms to Takahashi’s theory [8] and the result found for Ni_2_MnGa [12,13]. These relations held when the ratio of Ni to Ga and *e*/*a* were varied. *H* ∝ *M*^5^ behavior was also observed for MnSi [21] and Fe [22]. Therefore, Takahashi’s theory was again shown to be acceptable for use in analyzing magnetization in terms of itinerant electron ferromagnetism in Ni_2_MnGa system alloys. 

### 3.2. Basic Magnetic and Itinerant Spin Fluctuation Parameters and Generalized Rhodes–Wohlfarth Plot

In this subsection, we obtain the basic and spin fluctuation parameters and discuss itinerant magnetism by means of a generalized Rhodes-Wohlfarth plot of *p*_eff_/*p*_S_ versus *T*_C_/*T*_0_.

The induced magnetization *M* [8] (Equation (3.61)) is written as:

(6) $${\left(\frac{M}{M_{S}}\right)}^{4}=1.20\times 10^{6}\times \frac{T_{C}^{2}}{T_{A}^{3}}\times \frac{H}{M}$$

where *M*_S_ = *N*_0_*p*_S_*μ*_B_ represents a spontaneous magnetization in a ground state; *N*_0_ is a molecular number; *p*_S_ = *gS*, where *g* is the Landé’s *g*-factor and *S* is spin angular momentum; and *T*_A_ is the spin fluctuation parameter in wave number space (momentum space). *T*_A_ was obtained when experimental values were inserted into Equation (6), where the magnetic field *H* is in units of kOe and the magnetization *M* is in units of Am^2^/kg, which is equal to emu/g.

The spontaneous magnetic moment *p*_S_ (*μ*_B_) is expressed as:

(7) $$p_{S}^{2}=\frac{20T_{0}}{T_{A}}\times C_{\frac{4}{3}}\times {\left(\frac{T_{C}}{T_{0}}\right)}^{\frac{4}{3}}\;C_{\frac{4}{3}}=1.006089\cdots$$

where *T*_0_ is the width of the spin fluctuation spectrum in the energy scale. This appears as Equation (3.61) in Takahashi [8].

From Equation (7), *T*_0_ can be obtained using the formula:

(8) $$T_{0}=\frac{8147.2\times T_{C}^{4}}{T_{A}^{3}\times p_{S}^{6}}$$

Table 1 provides the measured spontaneous magnetic moment *p*_S_ and the characteristic temperatures *T*_C_, calculated *T*_A_, and *T*_0_ for Ni_2+*x*_MnGa_1__−*x*_. As for Ni_2_MnGa, the measured *p*_S_ of 3.93 μ_B_ is comparable to the theoretical band calculation result at the experimental lattice constant of the *L*2_1_ cubic austenite phase, *p*_S_, at 3.94 μ_B_ [23]. With increasing Ni fraction, the *p*_S_ value decreased. This behavior appears for Ni_2+*x*_Mn_1__−*x*_Ga [24] and Ni*_x_*Fe_1__−*x*_ Invar alloys [25]. *T*_0_ increased with increasing *x.* This is presumably because, in Equation (8), the right side varies with the sixth power of *p*_S_, so *T*_0_ varies even when *T*_A_ does not change. 

Takahashi also derived a formula [8], shown in Equation (3.47), for the relationship between *p*_S_, *T*_C_, *T*_0_, and the effective magnetic moment *p_eff_* as follows:

(9) $$\frac{p_{eff}}{p_{S}}\approx 1.4\times {\left(\frac{T_{0}}{T_{C}}\right)}^{\frac{2}{3}}$$

As for Ni_2_MnGa, *p_eff_* is 4.75 [24,26]. Equation (9) can be rewritten as:

(10) $$k_{m}=\left(\frac{p_{eff}}{p_{S}}\right)\times {\left(\frac{T_{C}}{T_{0}}\right)}^{\frac{2}{3}}$$

**Figure 5:**
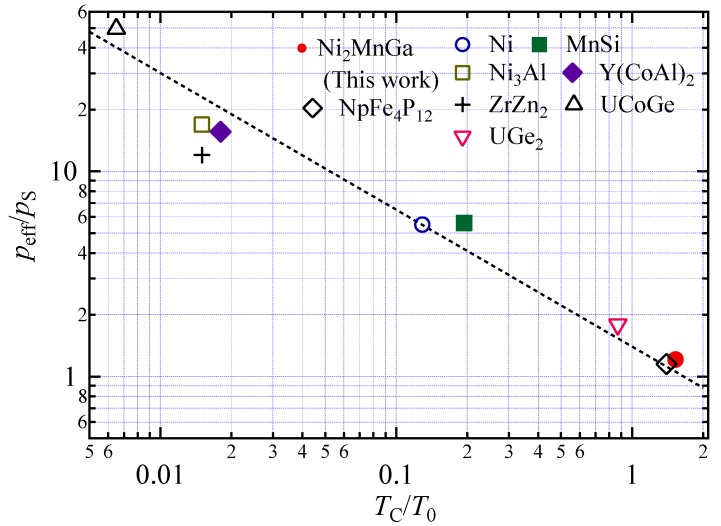
The generalized Rhodes-Wohlfarth plot (double logarithmic plot of *p*_eff_/*p*_S_ and *T*_C_/*T*_0_) for Ni_2_MnGa and other notable alloys and compounds. The dotted line indicates *k*_m_ = 1.4 as obtained from Equation (10).

When *k*_m_ is 1.4, Equation (10) is equal to Equation (9). For Ni_2_MnGa, a value of 1.61 for *k*_m_ was obtained by substituting *p*_S_, *T*_C_, and *T*_0_ from Table 1 and *p*_eff_ of 4.75 into Equation (10) [26]. The values of *k*_m_ for notable atoms, alloys, and compounds are Ni 1.41 [12], MnSi 1.88 [21], Ni_3_Al 1.06 [27], Y(Co_0.85_Al_0.15_)_2_ 1.08 [28], ZrZn_2_ 0.74 [29], UCoGe 1.74 [8], and UGe_2_ 1.61 [8]; these were calculated from the values listed in Table 2. Actinide 5*f* compound NpFe_4_P_12_ was also analyzed using the Takahashi theory and a *k*_m_ value of 1.44 was found [30]. Table 2 provides the *k*_m_ values and the magnetic moments and characteristic temperatures relating to spin fluctuation. Figure 5 is a plot of log(*p*_eff_/*p*_S_) versus log(*T*_C_/*T*_0_) for Ni_2_MnGa, Ni, and notable alloys and compounds using the data in Table 2. The dotted line indicates the line of Equation (10) when *k*_m_ is 1.4. Figure 5 clearly shows that the relation between *p*_eff_/*p*_S_ and *T*_o_/*T*_C_ can be explained by Equation (9). In Figure 3.3. in Takahashi [8], UGe_2_ had the largest value of *T*_C_/*T*_0_. In Figure 5 of this article, we added Ni, Ni_2_MnGa, and NpFe_4_P_12_. The *T*_C_/*T*_0_ value of Ni_2_MnGa was almost the same as that of NpFe_4_P_12_. The magnetic alloys and compounds that were analyzed by means of Equation (9) under the Takahashi theory were magnets with *T*_C_ values lower than room temperature. Notably, the ferromagnetic alloy Ni_2_MnGa, which has a *T*_C_ higher than room temperature, can be explained by Figure 5 and Equation (6).

The notable point from Table 2 and Figure 5 is that the *p*_eff_/*p*_S_ value of Ni_2_MnGa is smaller than those of other alloys and compounds. The effective moment *p*_eff_ was calculated from the Curie constant, C = *Nμ*_eff_^2^/3*k*_B_ = *Np*_eff_^2^*μ*_B_^2^/3*k*_B_ = *Nμ*_B_^2^*p*_C_(*p*_C_ + 2)/3*k*_B_. The term *p*_C_ refers to the effective moment deduced from the Curie constant *C*. The spontaneous magnetic moment *μ* is *p*_S_ (*μ*_B_) at 0 K. The *p*_c_/*p*_S_ was one for local moment ferromagnetism and was larger than one for itinerant ferromagnetism. For Ni_2_MnGa, *p*_eff_ was 4.75, as shown in Table 2; therefore, a *p*_c_ value of 3.85 was obtained from the equation *p*_eff_^2^ = *p*_C_(*p*_C_ + 2). Then, the *p*_C_/*p*_S_ value was 0.98. As a result, *p*_C_/*p*_S_ was a little smaller than one. Webster et al. compared the magnetic moment obtained by saturation magnetization measurement, *p*_sat_ = 4.17 [26]. Then, *p*_sat_/*p*_S_ was 0.92. In this work, the magnetization of Ni_2_MnGa in the magnetic field of 5.0 T at 5 K was 4.10 μ_B_/f.u. Therefore, *p*_sat_/*p*_S_ was 0.96. The Heusler compounds of CoMnSb and NiMnSb both possess the property of *p*_C_/*p*_S_ < 1 [31]. Ott et al. proposed a simple molecular field model considering both local moments and spin-polarized itinerant electrons to explain *p*_C_/*p*_S_ < 1 [31]. They introduced an enhanced temperature-independent Pauli susceptibility and explained that the Curie constant decreases if the interactions between local magnetic moments and holes is antiferromagnetic. Webster mentioned that in the paramagnetic phase, only the Mn atoms carry a magnetic moment [26]. It is supposed that in the paramagnetic phase, a large moment is induced by the electrons around the Mn atom at the Mn site. Conversely, at the Ni site, the spins fluctuate at high temperature in the paramagnetic phase. Therefore, it is supposed that the magnetic moment *p*_c_ at high temperature in a paramagnetic phase is smaller than the spontaneous magnetization *p*_S_ and the saturation moment *p*_sat_ at 5 K. 

### 3.3. Magnetization and Temperature Dependences Force Magnetostrictions

**Figure 6:**
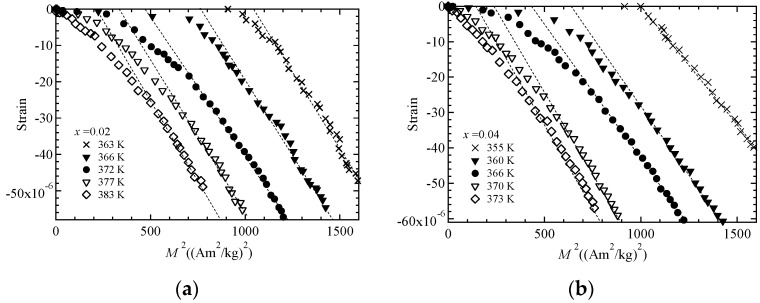
The *M*^2^ dependence of magnetostriction for (**a**) *x* = 0.02 and (**b**) *x* = 0.04. The dotted straight lines are included as a visual guide.

**Figure 7:**
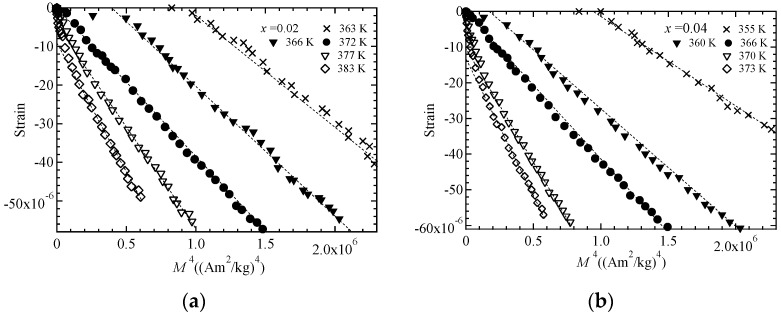
The *M*^4^ dependence of magnetostriction for (**a**) *x* = 0.02 and (**b**) *x* = 0.04. The dotted straight lines are included as a visual guide.

We recorded magnetostriction measurements to conduct an investigation into the magnetization dependence of forced magnetostriction. In our earlier study, the magnetostriction of Ni_2_MnGa was found to be proportional to the *M*^4^ of the magnetization and clearly passed through the origin at *T*_C_ [14]. In this study, we investigated Ni_2+*x*_MnGa_1−*x*_ (*x* = 0.02, 0.04) to check whether these relations held when the ratio of Ni to Ga and *e*/*a* were varied. We plotted figures of magnetostriction Δ*L*/*L* versus *M^δ^* for *δ* = 2.0 and 4.0. The result for *δ* = 2.0 indicates a relation under Moriya’s theory [1,15], and that for *δ* = 4.0 indicates a relation under Takahashi’s theory [8]. Figure 6 is a plot of magnetostriction Δ*L*/*L* versus *M*^2^ for *x* = 0.02 (Figure 6a) and *x* = 0.04 (Figure 6b). The dotted lines are fitted linear plots. For the magnetostriction at *T*_C_ indicated by the filled circles, the *M*^2^ linearity behavior was only observed for large magnetostriction and large magnetization area. Moreover, the dotted straight lines did not pass through the origin. These behaviors are comparable to the results for MnSi [15] and our former result for Ni_2_MnGa [13]. We also investigated Δ*L*/*L* versus *M*^4^ dependence, as shown in Figure 7. The plot of Δ*L*/*L* versus *M*^4^ indicates good linearity passing through the origin at *T*_C_, as indicated by the filled circles for both samples. Table 3 provides the coefficients *A* and *k* of the fitted linear plots given by the equation Δ*L*/*L* = *A* + *kM^δ^* for *δ* = 2 or 4 at *T*_C_. The standard deviations of the linear fitted lines at *T*_C_ for magnetostriction Δ*L*/*L* versus *M*^2^ and Δ*L*/*L* versus *M*^4^ are shown in Figure 6 and Figure 7, respectively, and are also listed in Table 3. The errors of the coefficient *k* were within ±2% for both values of *δ*. The proportions of the coefficient *A* and the magnetostriction at 5 T (Δ*L*/*L* ≃ −60 × 10^−6^), *y*_0_, were greater than 50% and less than 1.2% for *δ* = 2 and 4, respectively. This analysis indicates that the magnetostriction can be represented by the equation Δ*L*/*L* = *kM*^4^ at *T*_C_, as presented in Figure 7. As a result, the relation between magnetostriction and magnetization confirmed that the magnetostriction is proportional to the fourth power of the magnetization, as derived from Takahashi’s theory, even when the ratio of Ni to Ga and *e*/*a* were varied.

The magnetostrictions at 5 T were 50 × 10^−6^, 58 × 10^−6^, and 61 × 10^−6^ for *x* = 0.00, 0.02, and 0.04, respectively. With increasing *x*, the magnetostriction increased. In our former investigation of the magnetostriction of Ni_2_Mn_1−*x*_Cr*_x_*Ga (*x* ≤ 0.25), the magnitude of the magnetostriction increased when the premartensite transition temperature *T*_P_ and *T*_C_ were closer, as shown in Sakon et al. [16]. For Ni_2+*x*_MnGa_1−*x*_, the *T*_P_ values were 258 K, 265 K, and 275 K for *x* = 0.00, 0.02, and 0.04, respectively. The *T*_C_ values were 375 K, 372 K, and 366 K for *x* = 0.00, 0.02, and 0.04, respectively. With increasing *x*, the *T*_P_ shifted to higher temperatures and the *T*_C_ shifted to lower temperatures. We supposed that the magnetostriction of Ni_2+*x*_MnGa_1−*x*_ has the same properties as that of Ni_2_Mn_1−*x*_Cr*_x_*Ga.

**Figure 8:**
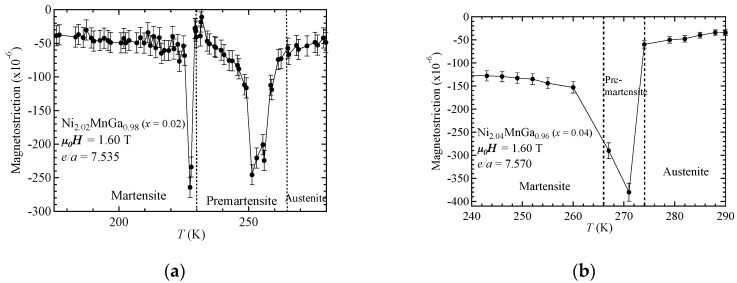
The temperature dependencies of the magnetostriction for (**a**) *x* = 0.02 and (**b**) *x* = 0.04.

**Figure 9:**
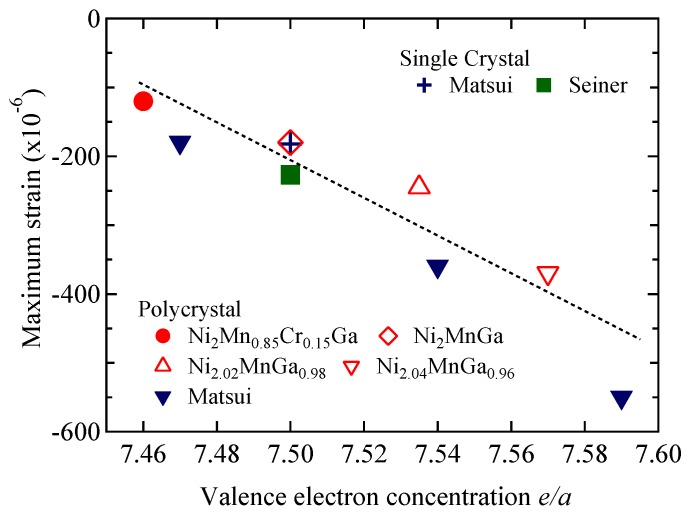
The *e*/*a* dependences of the maximum magnetostriction for Ni_2_MnGa-type alloys. Filled triangles: polycrystal, Matsui et al. [17,18]. Cross: single crystal, Matsui et al. [17]. Filled square: single crystal, Seiner et al. [33]. The dotted line is a fitted line.

Finally, we discuss the *e*/*a* dependences of the maximum magnetostriction around the premartensitic–austenitic transition for Ni_2_MnGa-type alloys. Around the premartensitic transition temperature *T*_P_, large magnetostriction has been observed [16,17]. Detailed explanations of the premartensitic transition and premartensite phase have been previously presented [16,17,18]. In our former investigation [16], we examined the magnetostrictions for Ni_2_Mn_1−*x*_Cr*_x_*Ga (*x* = 0.00, *e*/*a* = 7.50; *x* = 0.15, *e*/*a* = 7.46) around *T*_P_ and *T*_M_. With increasing *x*, *T*_P_ and *e*/*a* decreased; accordingly, the maximum value of the magnetostriction decreased. We assumed that if *e*/*a* increases, *T*_P_ and the magnetostriction increase. Matsui et al. experimentally investigated the Ni_2_MnGa-type alloys with *e*/*a* > 7.50 [17,18]. Among these alloys, Ni_51.7_Mn_24.3_Ga_24.0_ with *T*_P_ = 285 K and *e*/*a* = 7.59 showed large magnetostriction with strain 550 × 10^−6^ [17,18]. In this study, we decided to increase the concentration of Ni and decrease that of Ga because the *e*/*a* values of Ni and Ga are 10 and 3, respectively, in order to increase the *e*/*a* value of alloys to be above 7.50. Therefore, we prepared Ni_2+*x*_MnGa_1−*x*_ alloys with *x* = 0.02, producing *e*/*a* = 7.535, and *x* = 0.04, producing *e*/*a* = 7.570. Figure 8 shows the temperature dependencies of the magnetostriction at 1.6 T. The values were 250 × 10^−6^ and 380 × 10^−6^ for *x* = 0.02 and 0.04, respectively. Figure 9 shows the *e*/*a* dependences of the maximum magnetostriction for Ni_2_MnGa-type alloys. The maximum value of magnetostriction was almost proportional to the valence electron concentration per atom, *e*/*a*, and we also clarified the correlation between the magnetostriction and the *e*/*a*.

The softening of the lattice around *T*_P_ was investigated using ultrasonic measurements [32,33]. Seiner et al. investigated the magnetostriction around *T*_P_ for a single crystal of Ni_2_MnGa [33]. They suggested a model based on adaptive concept of premartensite, explaining the softening of *c*_44_ and apparent *c*′ stiffening prior to the martensitic transformation and discussed the magneto-elastic coupling effect by means of these magnetostriction, and ultrasonic measurements results under magnetic fields. This consideration only involves the softening of the elastic constant. Our experimental results indicate that the *e*/*a* and the magnetostriction are correlated and investigation by means of the itinerant electron magnetism is needed to better understand the fundamental origin of the magnetostriction. Future experimental and fundamental theoretical studies are needed to investigate the magneto-elastic coupling effect precisely, for example, with spectroscopy measurements for investigations of electron band structure and with itinerant electron magnetism theories.

## 4. Conclusions

Experimental investigations of the field dependence of magnetization and the relationship between magnetization and magnetostriction for Ni_2+*x*_MnGa_1−*x*_ (*x* = 0.00, 0.02, 0.04) alloy ferromagnets were performed in accordance with the self-consistent renormalization (SCR) spin fluctuation theory of itinerant ferromagnetism. In this study, we investigated the magnetization of and the magnetostriction on Ni_2+*x*_MnGa_1−*x*_ (*x* = 0.02, 0.04) to check whether these relations held when the ratio of Ni to Ga and *e*/*a* were varied. When the ratio of Ni to Ga varied, the valence electron concentration per atom, *e*/*a*, increased with increasing *x*. The magnetization results for *x* = 0.02 (*e*/*a* = 7.535) and 0.04 (*e*/*a* = 7.570) suggest that the critical index *δ* of *H* ∝ *M*^δ^ is around 5.0 at the Curie temperature *T*_C_, which is the critical temperature of the ferromagnetic–paramagnetic transition. This result confirms Takahashi’s spin fluctuation theory and the experimental results obtained for Ni_2_MnGa. The spontaneous magnetization *p*_S_ slightly decreased with increasing *x*. For *x* = 0.00, the obtained spin fluctuation parameter in *k*-space (momentum space) *T*_A_ and that in energy space *T*_0_ were 563 K and 245 K, respectively. The relationship between *p*_eff_/*p*_S_ and *T*_C_/*T*_0_ can be explained by Takahashi’s theory, where *p*_eff_ indicates the effective magnetic moments. We produced a generalized Rhodes-Wohlfarth plot of *p*_eff_/*p*_S_ versus *T*_C_/*T*_0_ values including those of other ferromagnets. The plot indicates that the relation between *p*_eff_/*p*_S_ and *T*_0_/*T*_C_ follows Takahashi’s theory. We also measured the magnetostriction for Ni_2+*x*_MnGa_1−*x*_ (*x* = 0.02, 0.04). At *T*_C_, the plot of the magnetostriction Δ*L*/*L* versus *M*^4^ showed proportionality and crossed the origin. These magnetization and magnetostriction results were analyzed in the context of Takahashi’s SCR spin fluctuation theory. Further, we investigated the magnetostriction at the premartensite phase, which is the precursor state to the martensitic transition. In Ni_2_MnGa system alloys, the maximum value of magnetostriction is almost proportional to *e*/*a*.

## Figures and Tables

**Table 1:** The spontaneous magnetic moment *p*_S_ and the characteristic temperatures *T*_C_, *T*_A_, and *T*_0_ for Ni_2+*x*_MnGa_1−*x*_.

*x*	*p*_S_ (μ_B_)	*T*_C_ (K)	*T*_A_ (K)	*T*_0_ (K)
0.00	3.93	375	563	245
0.02	3.79	372	566	288
0.04	3.64	366	567	345

**Table 2:** Basic magnetic parameters and *k*_m_, as obtained from Equation (10).

	*T*_C_ (K)	*p*_eff_ (μ_B_)	*p*_S_ (μ_B_)	*p*_eff_/*p*_S_	*T*_A_ (K)	*T*_0_ (K)	*T*_C_/*T*_0_	*k*_m_	Reference
Ni_2_MnGa	375	4.75 *	3.93	1.21	563	245	1.53	1.61	This work, [26] *
Ni	623	3.3	0.6	5.5	1.76 × 10^4^	4.83 × 10^3^	0.129	1.41	[12]
MnSi	30	2.2	0.4	5.3	2.08 × 10^3^	231	0.13	1.88	[21]
Ni_3_Al	41.5	1.3	0.075	17.3	3.09 × 10^4^	3.59 × 10^3^	0.016	1.06	[27]
Y(Co_0.85_Al_0.15_)_2_	26	2.15	0.138	15.6	0.726	1.41	0.018	1.08	[28]
ZrZn_2_	17	1.44	0.12	12	8.83 × 10^3^	321	0.053	0.74	[29]
UCoGe	2.4	1.93	0.039	49.5	5.92 × 10^3^	362	0.0065	1.74	[8]
UGe_2_	52.6	3.00	1.41	2.13	442	92.2	0.571	1.61	[8]
NpFe_4_P_12_	23	1.55	1.35	1.15	285	16.4	1.40	1.44	[30]

**Table 3:** The coefficients and standard deviations of the linear fitted plots obtained by means of the least squares method at *T*_C_ for the magnetostriction Δ*L*/*L* by the equation Δ*L*/*L* = *A* + *kM^δ^* for *δ* = 2 or 4, as shown in Figure 5 and Figure 6, respectively. Both *A* and *k* are constants.

	*δ* = 2		*δ* = 4	
*x*	0.02	0.04	0.02	0.04
*A*	3.60 × 10^−5^	1.65 × 10^−7^	3.29 × 10^−5^	−7.06 × 10^−7^
Standard deviation of *A*	±1.20 × 10^−6^ (3.3% of *A*)	±1.73 × 10^−7^ 105% of *A*)	±1.04 × 10^−6^ (3.2% of *A*)	±2.72 × 10^−7^ (38% of *A*)
*y*_0_ = *A*/(Strain at 5 T)	58%	0.3%	53%	1.2%
*k*	−7.62 × 10^−8^	−3.93 × 10^−11^	−7.58 × 10^−8^	−4.11 × 10^−11^
Standard deviation of *k*	±1.2 × 10^−9^ (1.5% of *k*)	±2.08 × 10^−13^ (0.5% of *k*)	±1.03 × 10^−9^ (1.4% of *k*)	±3.36 × 10^−13^ (0.8% of *k*)
